# Ultrashort Cationic Peptide Fmoc-FFK as Hydrogel Building Block for Potential Biomedical Applications

**DOI:** 10.3390/gels10010012

**Published:** 2023-12-22

**Authors:** Enrico Gallo, Carlo Diaferia, Sabrina Giordano, Elisabetta Rosa, Barbara Carrese, Gennaro Piccialli, Nicola Borbone, Giancarlo Morelli, Giorgia Oliviero, Antonella Accardo

**Affiliations:** 1 IRCCS SYNLAB SDN, Via Gianturco 113, 80143 Naples, Italy; enrico.gallo@synlab.it (E.G.); sabrina.giordano@synlab.it (S.G.); barbara.carrese@synlab.it (B.C.); 2 Department of Pharmacy, University of Naples “Federico II”, Via D. Montesano 49, 80131 Naples, Italy; carlo.diaferia@unina.it (C.D.); elisabetta.rosa@unina.it (E.R.); gennaro.piccialli@unina.it (G.P.); nicola.borbone@unina.it (N.B.); gmorelli@unina.it (G.M.); 3 Department of Molecular Medicine and Medical Biotechnologies, University of Naples “Federico II”, Via S. Pansini 5, 80131 Naples, Italy; giorgia.oliviero@unina.it

**Keywords:** peptide materials, hydrogels, cationic peptide, Fmoc-FF analogues, soft materials

## Abstract

Fmoc-diphenylalanine (Fmoc-FF) is a low-molecular-weight peptide hydrogelator. This simple all-aromatic peptide can generate self-supporting hydrogel materials, which have been proposed as novel materials for diagnostic and pharmaceutical applications. Our knowledge of the molecular determinants of Fmoc-FF aggregation is used as a guide to design new peptide-based gelators, with features for the development of improved tools. Here, we enlarge the plethora of Fmoc-FF-based hydrogelated matrices by studying the properties of the Fmoc-FFK tripeptide, alone or in combination with Fmoc-FF. For multicomponent matrices, the relative weight ratios between Fmoc-FFK and Fmoc-FF (specifically, 1/1, 1/5, 1/10, and 1/20 *w*/*w*) are evaluated. All the systems and their multiscale organization are studied using different experimental techniques, including rheology, circular dichroism, Fourier transform infrared spectroscopy, and scanning electron microscopy (SEM). Preliminary profiles of biocompatibility for the studied systems are also described by testing them in vitro on HaCaT and 3T3-L1 cell lines. Additionally, the lysine (K) residue at the C-terminus of the Fmoc-FF moiety introduces into the supramolecular material chemical functions (amino groups) which may be useful for modification/derivatization with bioactive molecules of interest, including diagnostic probes, chelating agents, active pharmaceutical ingredients, or peptide nucleic acids.

## 1. Introduction

Peptide aggregation behavior has a key role in biochemical research as the wide spectrum of supramolecular assemblies that may derive from the use of simple building blocks (short and ultra-short sequences of common and/or non-standard amino acids) offers inspiration for the generation of sophisticated devices to be applied in the biomedical sphere [1,2]. Among all peptide-based architectures (e.g., fibers [3], nanospheres [4], and micelles [5]), hydrogels (HGs) represent highly organized materials in which the network, generated by interconnected fibers, is able to retain, due to the hydrophilic portions of the amide skeleton, large amounts of water [6,7]. Thanks to their capabilities to accommodate active pharmaceutical ingredients (APIs) in their pores and to mimic the extracellular matrix because of their swollen structure, HGs have been successfully tested as drugs or contrast agents’ reservoirs and as matrices for tissue engineering applications [8,9]. The chemical, physical and mechanical properties of the hydrogel can be easily modulated by the opportune design of the peptide sequence, since the choice of the length, the amino acid lateral side and the insertion of aromatic or aliphatic groups at the N-terminus have a significant impact on its gelation capability and HG features [10,11,12,13]. Moreover, the multicomponent strategy, which consists of the combination of peptides with other building blocks (like different peptides or polymers), may also offer some advantages for generating hydrogels with improved mechanical behaviors [14,15]. The homodimer Fmoc-FF (N^α^-fluorenylmethoxycarbonyl-diphenylalanine) is one of the most studied peptide hydrogelators. Since the first analyses in 2006, this ultra-short sequence has been renowned for its displayed capability to form hydrogels in physiological conditions, combined with its synthetic accessibility [16,17]. Over the years, Fmoc-FF has represented the progenitor of a wide class of hydrogels, starting from analogues derived from the substitution of the fluorenyl moiety with other aromatic and non-aromatic groups to those made with changes to the peptide primary sequence [18]. Furthermore, a subclass of Fmoc-FF-derived materials is produced from the combination of Fmoc-FF with other chemical entities, according to the multicomponent strategy [19,20,21]. In this work, we describe the synthesis of a tripeptide originating from the addition of a lysine residue to the fluorenyl-protected diphenylalanine. The aminic group on the lysine side chain introduces a site accessible to its chemical functionalization, which at the same time, preserves the aromatic structure of the progenitor that is responsible for the aggregation. The derivatization of the Fmoc-FF base structure with an additional natural amino acid housing in its side chain, a positively charged functional group (Lys (K) or Arg (R)), has been recently tested by the group of He, Z [22,23]. The Fmoc-FFK and Fmoc-FFR tripeptides maintain the zwitterionic form, due to the fluorenyl end-capping of the N-terminus and give rise to different types of nanoarchitectures depending on the pH and the aliphatic lateral side. Moreover, the path of functionalization was performed by using Fmoc-FFR to immobilize gold nanoparticles (AuNPs) for nanocatalysis scopes, even though, in this case, the gelation capability of Fmoc-FFR was torn down by the alteration of the electrostatic balance along the peptide molecule after the derivatization of the NPs [24]. In our synthesis, the amidation of the C-terminus makes the tripeptide Fmoc-FFK cationic at a neutral pH, and no zwitterionic entity is accessible at any pH value. The gelation capability of this tripeptide was studied by exploiting the solvent switch method, which is one of the most used processes for hydrogel formulation and consists in the water dilution of a highly concentrated peptide solution in an organic solvent. Simultaneously, the possibility to generate multicomponent hydrogel matrices was also tested by combining Fmoc-FFK with Fmoc-FF at 1/1, 1/5, 1/10, and 1/20 weight/weight ratios. All the obtained matrices were fully characterized using a series of spectroscopic techniques, including fluorescence, Fourier transform infrared, circular dichroism, thioflavin T, and Congo red assays, scanning electron microscopy and optical microscopy. Rheological studies were also conducted to explore the mechanical properties of the resulting hydrogels. The biocompatibility of these matrices, together with their capability to support cell adhesion, was tested in vitro on HaCaT and 3T3-L1 cell lines.

## 2. Results and Discussion

### 2.1. Design and Synthesis of Fmoc-FFK

**Figure 1:**
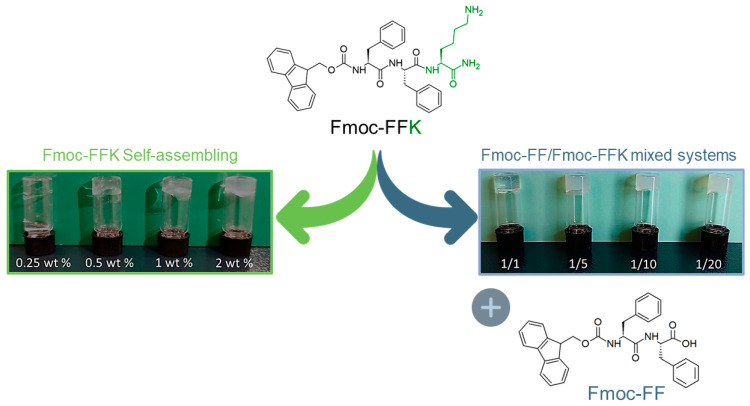
Schematic representation of the Fmoc-FFK tripeptide and the Fmoc-FF dipeptide. Inverted test tube of pure Fmoc-FFK hydrogel formulated at three different concentrations (0.5, 1.0, and 2.0 wt%) and the multicomponent Fmoc-FFK/Fmoc-FF hydrogels in which the two peptides are mixed in 1/1, 1/5, 1/10, and 1/20 (*w*/*w*) molar ratios.

The Fmoc-FFK tripeptide (see chemical formula in Figure 1) represents the elongated analogue of the well-known Fmoc-FF hydrogelator. Unlike the latter, Fmoc-FFK contains a potentially reactive group (*ε*-NH_2_), which can be opportunely used for functionalization with the desired moiety. The Lys residue has been positioned at the C-terminus which corresponds to the end of the aggregative group because the importance of the mutual steric proximity of both the Fmoc group and Phe residues for the self-assembly process and supramolecular organization has been demonstrated [25]. On the other hand, it is expected that any modification of the primary sequence could significantly alter the structural and functional properties of the material. Fmoc-FF generates a supramolecular assembly as consequence of an initial anti-parallel β-sheet secondary structure arrangement, with the anti-parallel π-stacking of the fluorenyl-protecting groups [26]. The additional lateral interlocking π–π interactions of four twisted anti-parallel β-sheets generates nanocylinders with an external diameter of ~3.0 nm.

The further adjacent self-associations of these structures allow for the formation of large, flat, twisted ribbons, which mutually block the water flow and form the hydrogel. The Fmoc-FFK tripeptide was synthetized according to solid-phase peptide synthesis (SPPS) protocols by using a Rink amide resin, which releases the peptide as an amide [27]. The capping of the carboxylic acid allows us to obtain only one potentially reactive group on the building block, thus reducing the reactive functions in the aggregate materials. Indeed, in a hypothetical post aggregative functionalization step, the lack of additional reactive groups, such as the carboxylic one, allows us to prevent collateral reactions, promoting a chemical control and increasing the selectivity of the reaction [28]. The crude peptide was purified via RP-HPLC chromatography, and its identity was assessed via an analytical HPLC characterization, ESI mass spectrometry, and ^1^H-NMR spectroscopy (see Figures S1 and S2). 

Initially, we evaluated the capability of the tripeptide to self-organize into a self-supporting hydrogel alone or in combination with the well-known hydrogelator Fmoc-FF at different weight/weight ratios (1/1, 1/5, 1/10 and 1/20 *w*/*w*). The hydrogel formation of ultrashort peptides can be generally induced using different approaches, like the pH-switch [29,30], the solvent switch [31,32], or the enzymatic one [33]. It is well documented that the choice of the method, as well as the solvent, the pH and the peptide concentration, can deeply affect the supramolecular architecture and the properties of the resulting material [34,35]. In our experiment, hydrogel formulation was achieved by using the solvent switch method, keeping the fraction of DMSO (ϕ_DMSO_ = 10%) unaltered. In this method, the gel formation is triggered by a physical stimulus, which is the addition of water to the peptide solution previously prepared in DMSO at a high concentration (100 mg/mL), with the formation of the peptide in a solvent–antisolvent mixture that induces the aggregation of monomers. After dilution in water, the sample is vortexed for few seconds to generate an opaque, uniform and metastable solution, then aged at room temperature. At the end of the preparation, the capability of the material to be self-supporting is macroscopically evaluated via the inverted test tube (Figure 1).

This test also allows for the determination of the critical gelation concentration (CGC), because the concentration is another important parameter for gel formation. The CGC for Fmoc-FFK was established by using samples at different concentrations between 0.25 and 2.0 wt% (Figure 1; green arrow) and estimated to be in the 0.5–1.0 wt% range. Instead, mixed hydrogels at different weight/weight ratios were prepared at 1.0 wt% only. Under these conditions, the inverted test tube confirmed the gel state for all the samples. During the preparation of multicomponent hydrogels, we qualitatively observed a different gelation time as a function of the chosen ratio. To quantitatively estimate the kinetics of gelation, the decrease in the refractive index related to the transition of the metastable gel solution from opaque to limpid was measured via UV-vis spectroscopy. By plotting the absorbance at 600 nm (the long-range channel) as a function of time, a decrease in the optical density (OD) can be observed, which corresponds to the supramolecular organization of peptide building blocks into fibers having a size lower than that of the particles scattering the light. The gelation times (G_ts_) were graphically extrapolated from the graph considering the flex concavity of the profiles (Table 1, Figure S6). Surprisingly, the gel kinetics significantly increase from 5 to 216 min with an increase in the Fmoc-FF ratio in the mixture. The capability of pure and mixed matrices to incorporate water (swelling) was estimated by performing a swelling test; the data were reported in terms of the swelling percentage (q). According to Equation (1), the *q* values are calculated by putting the gel in contact with water and determining the increase in weight that occurs. The swelling percentage ranged between 32 and 37% for all the samples (Table 1) and resulted to be slightly higher than the percentage determined for the pure Fmoc-FF hydrogel (29%) [36,37]. This value is in agreement with the ones observed for similar Fmoc-FF/tripeptide mixed systems. The relatively low swelling percentage may be due to the high hydrophobic component of the sequence, as a substantial aromatic part is present and may contribute to the low amount of entrapped water. Indeed, the addition of a more hydrophilic component results in an increase in the swelling ratio when compared to that of the pure Fmoc-FF (see Table S1) [36].

### 2.2. Scanning Electron Microscopy (SEM) Characterization

**Figure 2:**
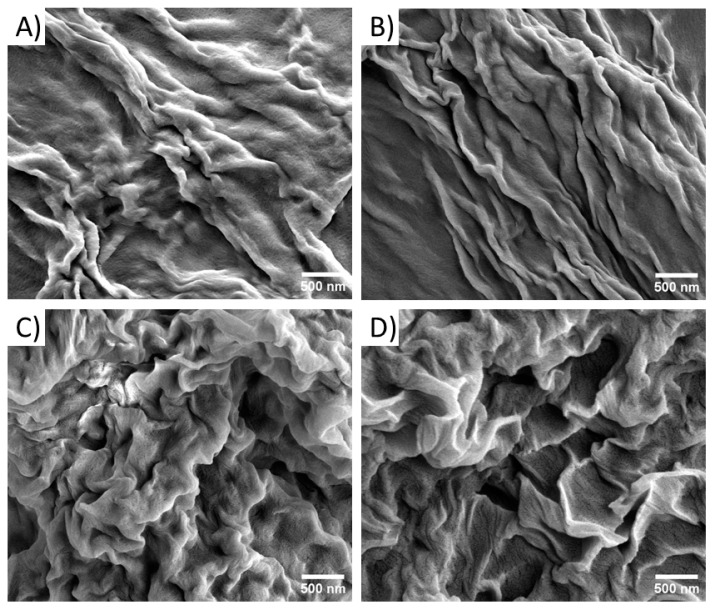
Selected microphotos of mixed xerogels: Fmoc-FFK/Fmoc-FF at 1/1 (**A**), 1/5 (**B**), 1/10 (**C**), and 1/20 (**D**). Scale bar: 500 nm.

Morphological investigations on the pure and mixed hydrogels were carried out via scanning electron microscopy. Microphotos were acquired on xerogels prepared on aluminum stubs. The characterization performed on xerogels of pure Fmoc-FFK hydrogels at different concentrations (0.5, 1.0, and 2.0 wt%) shows that only the sample at 2.0 wt% had well-defined organization in its entangled fibers (Figure S4). On the contrary, no fibrillary networks, typical of hydrogel matrices, can be detected in the samples prepared at the other two lower studied concentrations (0.5 and 1.0 wt%). This result is in good agreement with the CGC previously found by the inverted test tube method. Instead, all the mixed hydrogels exhibit a similar morphology independently from the ratio between the two peptides (Figure 2).

### 2.3. Secondary Structural Characterization

**Figure 3:**
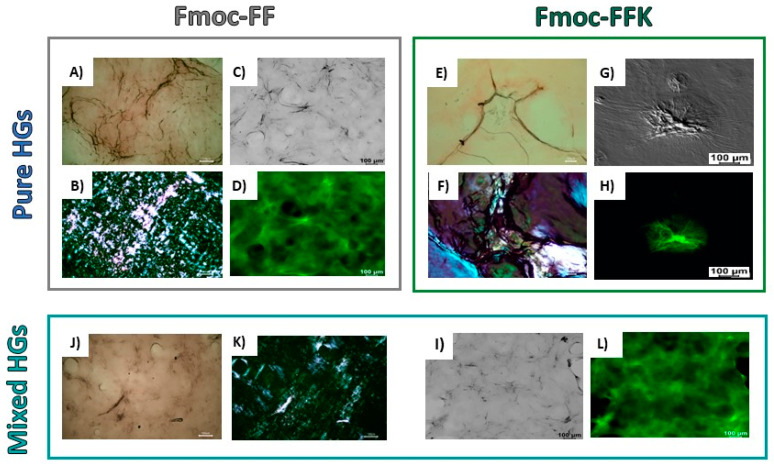
Birefringence CR assay and ThT assays for xerogels prepared by pure Fmoc-FF and Fmoc-FFK HGs (1.0 wt%) and mixed Fmoc-FFK/Fmoc-FF HGs (1.0 wt%, 1/5 *w*/*w*). Polarized optical microscopy images of air-dried samples stained with Congo red solution under bright-field microscopy (**A**,**E**,**J**) and between crossed polarizers (**B**,**F**,**K**). Confocal images of xerogels, stained with 50 μmol/L ThT solution, under bright-field microscopy (**C**,**G**,**I**) and in the green spectral region (GFP). λ_exc_ = 450 nm; 460 nm < λ_em_ < 600 nm (**D**,**H**,**L**).

**Figure 4:**
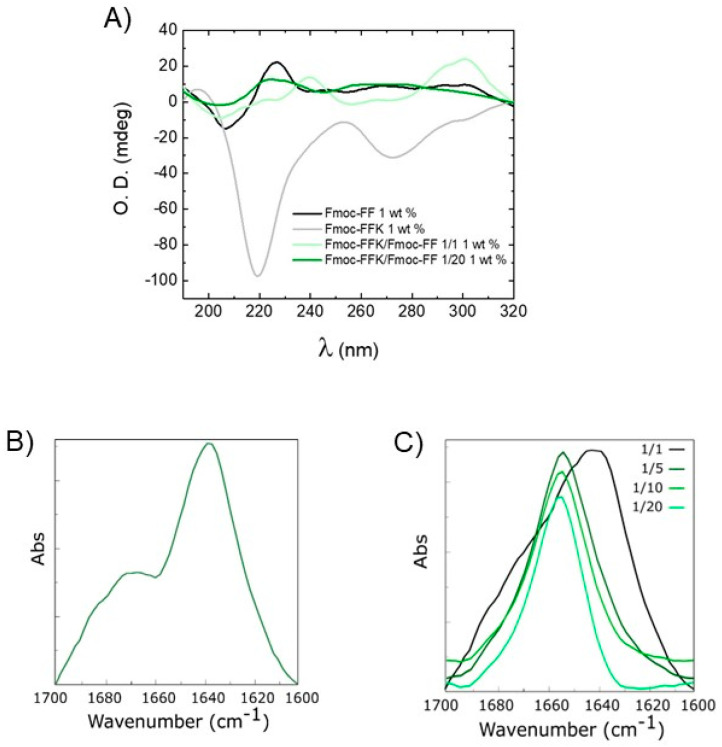
Secondary structure characterization of mixed hydrogels via CD and FTIR. (**A**) CD spectra in optical density of hydrogels at 1.0 wt%: Fmoc-FF (black line), Fmoc-FFK (grey line), Fmoc-FFK/Fmoc-FF (1/1) (light green line), and Fmoc-FFK/Fmoc-FF (1/20) (dark green line). FTIR spectra of pure Fmoc-FFK HGs (**B**) and mixed Fmoc-FFK/Fmoc-FF (**C**) at 1.0 wt% at all the weight/weight ratios.

The secondary structural organization of peptide units into the pure and mixed hydrogels was studied by using a combination of optical (microscopy and confocal) and spectroscopic (circular dichroism, Fourier transform infrared and fluorescence) techniques. A preliminary characterization of the arrangement of peptide sequences in the hydrogels was performed on samples in the solid state. Xerogels, prepared on glass from the corresponding hydrogels, were screened using two well-assessed assays (birefringence of Congo red and thioflavin T), which are commonly employed for revealing the presence of amyloid-like structures. Indeed, it has been demonstrated that both the CR and ThT undergo to a modification of their spectroscopic properties when they interact with β-sheet structures, with the appearance of an apple-green birefringence under cross-polarized light for the azoic CR dye and a fluorescence emission around 480 nm for ThT. From an inspection of Figure 3, it can be observed that both the pure and mixed Fmoc-FFK/Fmoc-FF xerogels stained with CR and ThT are positive to the assays, thus suggesting a β-sheet arrangement of peptides in the supramolecular matrices. The data gathered from spectroscopic techniques, like CD and FT-IR, can be combined to obtain information about the molecular organization within the hydrogel matrices. By comparing the shape of the CD spectra belonging to the mono-component Fmoc-FF and Fmoc-FFK HGs and the multi-component Fmoc-FFK/Fmoc-FF systems (with ratios of 1/1 and 1/20), provided in Figure 4A, it can be deduced that each matrix is affected by different organization modes. The secondary structure of Fmoc-FF has been widely analyzed and discussed [38]. The positive signal around 195 nm and the negative one centered at 206 nm are ascribable to π→π* α-helix transitions. A super-helical arrangement of the phenylalanine residues is deduced by the 236 nm-centered broad band, attributed to n→π* transitions.

By looking at the CD profile of the self-assembling Fmoc-FFK HG at the same 1 wt% peptide concentration, whose shape is analogous to that at the 2.0 wt% concentration (Figure S5), a β-sheet organization is clearly inferred by the positive signal at 196 nm (π→π* transitions) and the negative one at 219 nm (n→π* transitions). Moreover, the negative band in the spectral region of the Fmoc moiety (centered at 276 nm) is attributed to the π–π stacking of aromatic fluorenyl rings [39]. In the 1/1 multi-component HG, the weak negative bands at 204 and 226 nm suggest the occurrence of a negligible α-helix organization, while the more pronounced positive peak at 238 nm suggests an antiparallel orientation of β-sheets [40]. The π–π* transitions of the fluorenyl are responsible for the generation of a positive signal at 301 nm [41]. By comparing the CD signature of this 1/1 mixed matrix with the one obtained by the arithmetic sum of the spectra of the two components alone, a co-assembly aggregation is inferred, as the two cited original and mathematical-derived spectra are different [42]. This ability of the components to arrange together gives rise to novel architectures, resulting from the interactions of the different building blocks with each other. This cannot be said for the Fmoc-FFK/Fmoc-FF 1/20 mixed matrix, for which the CD behavior seems to be driven by the Fmoc-FF, as the same previously cited peaks for the dipeptide have been noted. Even for the HGs in which the amount of Fmoc-FF is not so preponderant (at ratios of 1/5 and 1/10), the CD profiles follow the signature of Fmoc-FF, and thus, a similar secondary organization occurs (Figure S5). As the spectrum obtained by the sum of the optical density values of the two single components is similar to the one obtained by the analysis of the 1/20 HG, it can be deduced that in this kind of double-composed HG a self-sorting organization occurs, which means that the different building blocks are able to selectively recognize their mutual counterparts by generating mono-component supramolecular assemblies, interacting with the other singularly organized systems in the environment at a superior level [43]. To support the CD spectroscopic analyses, Fourier transform infrared (FT-IR) measurements were taken. The peptide signals in the amide I region (1700–1600 cm^–1^), due, for the 80%, to the C=O stretching vibrations, can reveal the secondary structure arrangements [44]. For the Fmoc-FFK and the Fmoc-FFK/Fmoc-FF 1/1 mixed matrices, the band around 1640 cm^–1^ clearly indicates a β-sheet organization, as previously assessed via CD spectroscopy. The supplemental signal at 1672 cm^–1^ is attributable to the presence of TFA counterions (Figure 4B). Contrarily, for the mixed HG with a 1/20 ratio, the band centered around 1660 cm^–1^, generally attributed to α-helix or super helical conformations, confirms the predominance of the Fmoc-FF structuration [21,45]. The same band was detected for the 1/5 and 1/10 ratios (Figure 4C). The progressive modification of the supramolecular arrangement is reinforced by a deconvolution analysis using weighted percentages of each mixed hydrogel (Table S2), indicating the increase in α-helix/super helical conformations with the increase in Fmoc-FF percentage in the mixed matrices. 

### 2.4. Rheological Characterization

**Figure 5:**
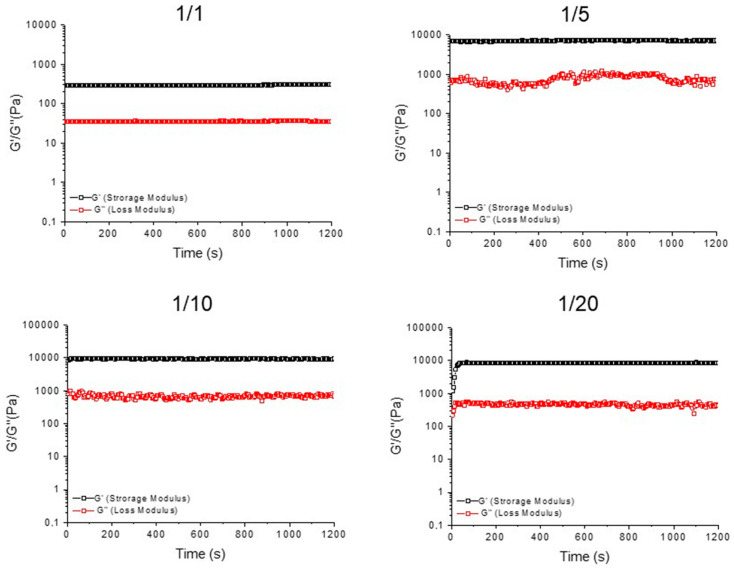
Time sweep (20 min) for mixed hydrogels Fmoc-FFK/Fmoc-FF at three different weight/weight ratios (1/1, 1/5, 1/10, and 1/20). The rheological analysis is reported in terms of G′ (storage modulus) and G″ (loss modulus).

To confirm the gel status of all the samples, the storage modulus (G′) and the loss modulus (G″) were evaluated by performing a rotational rheological analysis. The time sweep oscillatory measurements (20 min, 1.0 Hz, and 0.1 % strain; Figure 5 and Figure S6) were carried out on the preformed samples, after the acquisition of both the frequency (0.1 < ν < 100 Hz; Figures S7A and S8A) and strain sweeps (0.1 < ω < 100%; Figures S7B and S8B). As expected for hydrogels, the G′ value is higher than the G″ one (Table 1), thus analytically confirming that all the studied samples are in the gel state. 

From the inspection of the values in Table 1, it can be pointed out that the self-assembled Fmoc-FFK hydrogel has a very soft nature at all the studied concentrations (G′ = 5.8, 15.4, and 24.3 Pa for 0.5, 1.0, and 2.0 wt%, respectively). Additionally, a monotone increase in both G′ and G″ with the frequency was detected for the pure Fmoc-FFK matrices (Figure S9A). This trend is independent from the gel concentration, indicating an increase in the elastic response because of the decreasing dissipative stress time. Moreover, no creep oscillation value is detected for the pure Fmoc-FFK hydrogels (Figure S9B). The inclusion of the increasing amount of Fmoc-FF peptide into the formulation allows us to gradually improve the mechanical properties of the gel, with a significant rigidification of the matrix for the 1/10 and 1/20 ratios (G′ = 9800 and 9412 Pa). The existence of a strong matrix, with a prominent viscoelastic nature, is also confirmed by the loss tangent (tanδ =G″/G′) value, which is 0.0526 for Fmoc-FFK/Fmoc-FF (1/20, *w*/*w*). This increase in the mechanical features, which are well documented for other multicomponent matrices, seems to be related to the ability of the Fmoc-FF building block to govern and drive the gelation process, reducing the probable electrostatic repulsion in pure Fmoc-FFK gels [46]. These electrostatic repulsions may justify the low mechanical response of Fmoc-FFK hydrogels, as evidenced by the lower G′ values for multicomponent matrices with respect to the Fmoc-FF one (G′ = 23,160 Pa at 1.0 wt% and 1.0 Hz; rheological characterization reported in Figure S10).

### 2.5. Loading and Release of Naphthol Yellow S

The capacity of these hydrogels to retain drugs and to serve as reservoirs for controlled drug release was tested by encapsulating NYS in the matrices and evaluating its release over time. NYS is a water-soluble disodium salt of 5,7-dinitro-8-hydroxynaphthalene-2-sulfonic acid, a histological dye, herein used as drug model, easily shown via UV-vis spectroscopy. A total of 0.011 mol/L NYS was encapsulated within the hydrogel network during the rehydration step of the solvent switch formulation method. The gelation kinetics and the matrices’ homogeneity were found to be not affected by the drug’s inclusion. No syneresis was observed after the quantitative loading of the dye.

The release kinetics of NYS over 72 h are reported in Figure S11. The two more rigid Fmoc-FFK/Fmoc-FF 1/10 and 1/20 HGs were chosen to be compared with the mono-component Fmoc-FF precursor. Despite the significant difference in rigidity, Fmoc-FF and Fmoc-FFK/Fmoc-FF 1/20 exhibit a similar release behavior, with a NYS release around 25% of the initial amount over 72 h. On the other hand, a higher release (~35%) is observed for Fmoc-FFK/Fmoc-FF at the ratio of 1/10, which has the same rigidity as the other mixed hydrogel and a higher positive charge content. These results point out that additional factors, with respect to stiffness and electrostatic interactions, can play a role in drug retention. 

A plausible explanation is that the escape tendency of the dye from HG could be affected by the different hydrophilic/hydrophobic natures of the matrix, which cause different levels of water accessibility. 

### 2.6. Cytotoxicity and Cell Adhesion Assays

The cytotoxicity of the multicomponent hydrogels was evaluated in vitro on HaCaT (human fibroblasts) and 3T3-L1(mouse preadipocytes) cell lines by using an MTS assay, incubating the cells with the conditioned media up to 72 h. It was not possible to carry out the experiment for the 1.0 wt% or the 2.0 wt% self-assembled Fmoc-FFK hydrogels, as their high softness made them solve in few times in DMEM when incubated with the medium. No significant change in cell morphology was observed to be induced by the hydrogel treatment compared to the control. 

**Figure 6:**
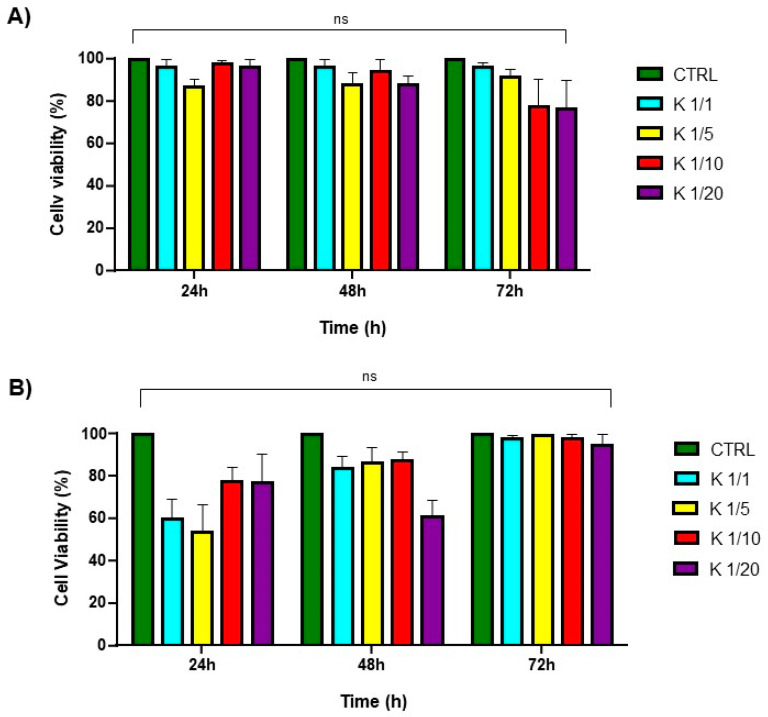
Cell viability assay. MTS assay of HaCaT (**A**) and 3T3-L1 (**B**) cell lines treated for 24, 48, and 72 h with hydrogel’sconditioned medium. ns: not significant versus control cells.

**Figure 7:**
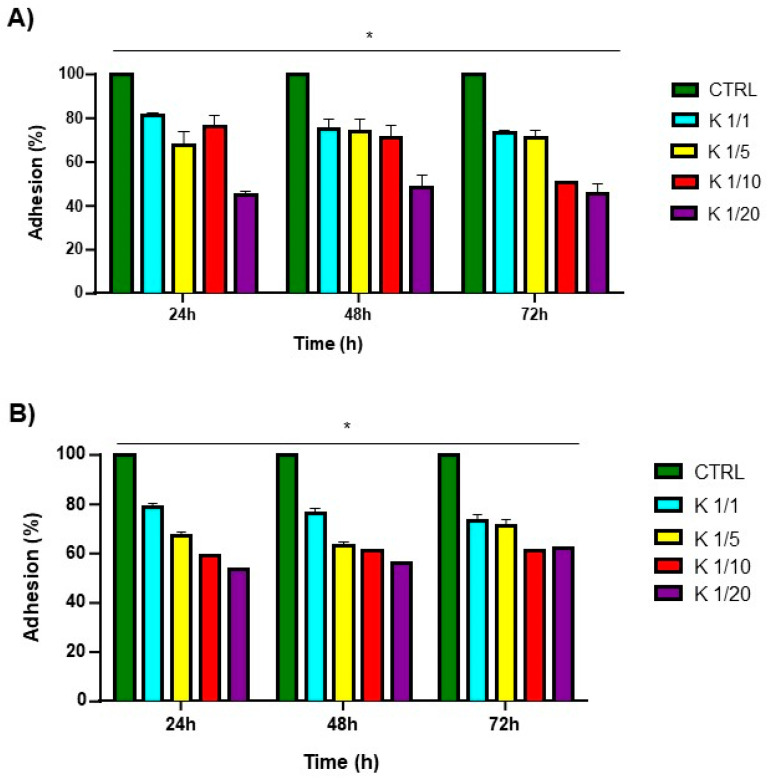
Adhesion test of HaCaT (**A**) and 3T3-L1 (**B**) cell lines for 24, 48, and 72 h on indicated mixed hydrogels. * *p* < 0.05, versus control cells.

As previously observed for Fmoc-FF hydrogels, a maximum toxicity was reached after 24 h (Figure 6), while the cell survival percentage had increased at the subsequent observation times [47]. This effect may be ascribed to a temporary cycle arrest at the S phase, followed by a complete recovery of the cells (negligible or no toxicity compared to the control for all the explored ratios) after 48 and 72 h (Figure 6). The capability of the matrices to work as scaffolds able to support cell adhesion and proliferation was evaluated by culturing HaCa T and 3T3-L1 cell lines on pre-casted hydrogels. The adhesion efficiency was measured up to 72 h after seeding and the results are expressed in terms of the percentage of adhered cells to the hydrogel compared to that of control cells (without the hydrogel). As shown in Figure 7, these adhesion percentages were found to be 73%, 71%, 50%, and 39% for the HaCaT cells and 74%, 72%, 58%, and 47% for the 3T3-L1 cells cultured on Fmoc-FFK at ratios of 1/1, 1/5, 1/10, and 1/20, respectively. An inverse correlation between the hydrogel rigidity and adhesion efficiency may be observed, since softer hydrogels seem to be better scaffolds for both of the tested cell lines. 

## 3. Conclusions

In the last few years, short and ultrashort peptides have been identified as potential building blocks for the formulation of biocompatible materials, like nanotubes, fibers, nanospheres, and hydrogels. The main advantages offered by peptides are their low cost and the possibility to opportunely change the primary sequence to modulate their structure and function. Indeed, it is observed that the modification of the amino acid sequence can deeply alter the intermolecular interactions occurring in the supramolecular structure, with a consequent change in the structural properties that also affects the features of the material, like its mechanical rigidity, elasticity, and water content. These modifications in turn allow us to fabricate supramolecular systems, differing in morphology and performances, with a broad range of biomedical applications [48,49]. For instance, soft hydrogels are principally employed as injectable implants, whereas rigid ones are generally preferred for the regeneration of hard tissues, such as bone and cartilage. In this context, research on novel building blocks that are easy to synthetize is trending. The Fmoc-FF moiety represents a good starting point for the design of novel hydrogelators. Additionally, due to its high accessible gelation procedure (solvent switch), the combination of Fmoc-FF with other peptide sequences or polymers can allow for the development of novel materials. According to our findings, it can be observed that the Fmoc-FFK monomer, in which Fmoc-FF has been modified with a lysine residue at its C-terminus, is able to self-assemble and gel above the critical concentration of 1.0 wt%. The resulting self-supporting hydrogel exhibits a soft behavior with a G′ value (24 Pa) that is very low with respect to that of the parental Fmoc-FF derivative (G′ = 23,160 Pa). Considering that the two peptides share the same N-terminus sequence, their significantly different stiffness values are to be attributed to the positive charge that the Lys bears on the ε-NH. On the other hand, the co-aggregation of the novel Fmoc-tripeptide with Fmoc-FF at different weight/weight percentages (1/1, 1/5 1/10 and 1/20) allows us to produce matrices with enhanced mechanical properties with respect to Fmoc-FFK alone. The multicomponent hydrogels, exhibiting a similar structural organization and morphology with respect to those of a pure Fmoc-FF hydrogel, could potentially serve as platforms for post-gelation derivatization with bioactive molecules that bear a primary amine for diagnostic or therapeutic purposes.

## 4. Materials and Methods

### 4.1. Materials and Methods

Rink amide MBHA (4-methylbenzhydrylamine) resin, coupling reagents, and protected N^α^-Fmoc-amino acid derivatives were supplied by Calbiochem-Novabiochem (Läufelfingen, Switzerland). Bachem (Bu-bendorf, Switzerland) provided the powder of Fmoc-FF peptide. Unless otherwise indicated, all other chemical items were used as specified by the manufacturers and were commercially accessible from Merck (Milan, Italy), Fluka (Bucks, Switzerland), or LabScan (Stillorgan, Dublin, Ireland). Samples and peptide-based hydrogels were prepared by weight using dimethyl sulfoxide (DMSO) and double distilled water, respectively. The crude peptides were purified via preparative reversed-phase high-performance liquid chromatography (RP-HPLC) on a Phenomenex (Torrance, CA, USA) C18 column. The LC8 Shimadzu HPLC system (Shimadzu Corporation, Kyoto, Japan) was employed, equipped with a UV Lambda-Max Model 481 detector. H2O/0.1% trifluoroacetic acid (TFA) (A) and CH3CN/0.1% TFA (B) were used as elution solvents. The concentrations were increased from 20% to 90% in 30 min at a flow rate of 20 mL min^–1^. The purity of the products was assessed by analytical reversed-phase high-performance liquid chromatography (RP-HPLC) analysis performed by using Finnigan Surveyor MSQ single quadrupole electrospray ionization (Finnigan/Thermo Electron Corporation San Jose, CA, USA), with a C18-Phenomenex column eluting with H2O/0.1% TFA (A) and CH3CN/0.1% TFA (B) from 20% to 90% over 25 min at a flow rate of 1 mL min^–1^. The identity of peptides was assessed by mass spectrometry using a LTQ XL Linear, ion trap mass spectrometer, ESI source (Finnigan/Thermo Electron Corporation, San Jose, CA, USA).

### 4.2. Solid-Phase Peptide Synthesis 

Standard solid-phase peptide synthesis (SPPS) protocols were employed to synthesize the Fmoc-FFK peptide via an Fmoc/tBu strategy [50]. Essentially, *N*,*N*-dimethylformamide (DMF) was used as swelling solvent for Rink amide MBHA resin (substitution grade: 0.72 mmol g^−1^) for 45 min. Fmoc cleavage was achieved by treating the solid support with a 20% *v*/*v* piperidine solution in DMF. Each amino acid was coupled by allowing the resin to react for 45 min with a two-fold molar excess of 1-hydroxybenzotriazole (HOBt) and HBTU (hexafluorophosphate benzotriazole tetramethyl uronium) (HBTU), as well as a four-fold molar excess of di-isopropylethylamine (DIPEA) in DMF as the reaction solvent. Using a TFA/triisopropylsilane (TIS)/H_2_O (92.5/5.0/2.5 *v*/*v*/*v*) solution, the crude peptide was removed from the resin. The peptide was then precipitated in cold ether and freeze-dried three times. The crude peptide was found to be over 80% pure.

### 4.3. Fmoc-FFK Characterization

t_R_ = 18.31 min, MS (ESI+): *m*/*z*: calcd. for C_39_H_42_N_4_O_6_: 661.5 [M+H]^+^; found: 662.5; ^1^H NMR (700 MHz, DMSO) δ 8.15 (d, *J* = 7.9 Hz, 1H), 8.06 (d, *J* = 8.2 Hz, 1H), 7.95–7.86 (m, 3H), 7.62 (d, *J* = 7.5 Hz, 1H), 7.59 (dd, *J* = 11.8, 8.1 Hz, 2H), 7.46–7.38 (m, 3H), 7.32 (td, *J* = 7.4, 1.1 Hz, 1H), 7.30–7.22 (m, 10H), 7.21–7.13 (m, 3H), 7.11–7.05 (m, 1H), 4.56 (td, *J* = 8.4, 4.9 Hz, 1H), 4.27–4.16 (m, 3H), 4.15–4.07 (m, 2H), 3.07 (dd, *J* = 14.0, 4.9 Hz, 1H), 2.92 (dd, *J* = 13.8, 3.9 Hz, 1H), 2.86 (dd, *J* = 14.0, 9.1 Hz, 1H), 2.75 (t, *J* = 7.7 Hz, 3H), 2.70 (dd, *J* = 13.8, 10.8 Hz, 1H), 1.68 (td, *J* = 10.9, 5.4 Hz, 1H), 1.60–1.46 (m, 4H), 1.30 (tt, *J* = 16.4, 6.9 Hz, 3H).

### 4.4. Hydrogels Formulation

The DMSO/H_2_O solvent switch approach was used to generate self-assembled hydrogels at four distinct weight/weight percentages: 0.25 wt%, 0.50 wt%, 1.0 wt%, and 2.0 wt%. A concentration of 1.0 weight percentage (10 mg mL^−1^) of mixed hydrogels was prepared using the DMSO/H_2_O solvent switch method. For mixed hydrogels, the ratios of 1/1, 1/5, 1/10, and 1/20 *w*/*w* were examined. The stock solution for each peptide was prepared in DMSO (100 mg mL^−1^), mixed, vortexed, and then rehydrated with water. To improve sample homogeneity throughout the rehydration procedure, the solutions were also stirred for two seconds. The macroscopic assessment of hydrogel formation was conducted through the inverted tube test.

### 4.5. Hydrogel Swelling Studies

Hydrogel swelling ratios were determined by incubating each hydrogel sample (1.0 wt%, V = 300 µL) overnight at 25 °C upon the addition of 900 µL of doubly distilled water. Immediately after the removal of excess water, fully swollen hydrogels were weighed (*Ws*). Following freeze-drying, the hydrogels were weighed once again (*Wd*). The equation below states that the swelling behavior can be represented as the swelling ratio (*q*), which is the ratio between the weight of the swollen sample (*Ws*) and the weight of the freeze-dried hydrogel (*Wd*).

$$q=\frac{\left(Ws-Wd\right)}{Wd}\%$$

### 4.6. Circular Dichroism (CD) Studies 

Jasco J-1500-150 spectropolarimeter equipped with a Jasco MCB-100 Mini Water Circulation Bath thermal controller unit (Peltier device) was used to gather the far-UV CD spectra of pure and mixed hydrogels, using a 0.2 mm quartz cell at 25 °C. Sample spectra were collected from 320 to 190 nm at various concentrations. Additional experimental parameters included the following: scan speed = 20 nm min^–1^, sensitivity = 200 mdeg, time constant = 1 s, and bandwidth = 1 nm. Each spectrum was obtained by averaging three scans and correcting for the blank.

### 4.7. FT-IR Spectroscopy

The pure and mixed hydrogels (1.0 wt%) were examined through FT-IR spectroscopy. The spectra were obtained by using a Ge single crystal at a resolution of 4 cm^–1^ in attenuated total reflection mode on a Jasco FT/IR 4100 spectrometer (Easton, MD, USA). Every sample was scanned 100 times at a rate of 2 mm s^–1^ on a KBr background. The instrument integrated software immediately returned the amide I deconvolutions (in the 1600–1700 cm^–1^ area) as emissions after they were collected in transmission mode. A quantitative multivariate percentage analysis for secondary structure estimation (SEE) was achieved via method of principal component regression (PCR) with the Jasco SEE^®^ dedicated software (Easton, MD, USA).

### 4.8. Thioflavin T (ThT) Spectroscopic Assay

ThT assay was used to assess the aggregation behavior of xerogels made by mixing hydrogels. Rapid interaction between thioflavin T and β-aggregated peptides results in enhanced emission at 482 nm [51]. Using the solvent switch approach, hydrogels were formulated by first diluting the peptide stock solution in DMSO (100 mg mL^–1^) and then by adding water. Following the preparation of gels, samples were placed onto a clean glass coverslip, allowed to air dry, and then stained with 50 μL of a 50 µmol L^–1^ ThT solution. To prevent any corruption of the dried xerogels, the excess ThT solution on the samples was removed using filter paper. The dried stained films were examined in the GFP (green fluorescent protein) spectral region, with λ_exc_ = 488 nm and λ_em_ = 507 nm, under bright-field illumination. Fluorescence emission spectra were recorded between 460 and 600 nm after samples were excited at 450 nm. To acquire immunofluorescence pictures, scale bars were recorded at a resolution of 100 μm for each photograph, by means of a Leica MICA microhub fluorescent microscope with a 10× magnification. 

### 4.9. Birefringence CR Assay

Owing to its ability to bind amyloid-like fibers, CR dye is frequently used to identify β-aggregated peptides [52]. To prepare the samples, ≈40 μL of each preformed peptide hydrogel was drop-cast onto a glass microscope slide. HGs were immediately ready for analysis, as previously mentioned. A total of 2 µL of a saturated CR solution dissolved in H_2_O/ethanol (80/20, *v*/*v*) and saturated with NaCl was added to the hydrogels during the formulation step. Subsequently, the hydrogel was placed onto a glass microscope slide and air-dried overnight at room temperature. The obtained dried films were examined using an Optech BM80 Pol microscope (Milan, Italy) for both crossed polars and bright-field lighting.

### 4.10. Rheological Studies 

Freshly preformed hybrid hydrogels (500 μL) underwent rheological assessments employing 1.5 cm diameter flat-plate geometry (PU20-PL61) and a rotational controlled-stress rheometer (Malvern Kinexus, UK). Each measurement was carried out in a humidity chamber with a 1.0 mm gap interval at 25 °C. Initial optimization parameters were carried out using strain sweep (0.1–100%) and oscillation frequency (0.1–100 Hz). Subsequently, an oscillatory time-sweep study lasting 15 min was carried out at 25 °C, 0.1% strain, and 1.0 Hz frequency. Rheological profiles were plotted as storage or elastic modulus (*G*′) and shear loss or viscous modulus (*G*″). The profiles were reported in Pascal (Pa).

### 4.11. Scanning Electron Microscopy (SEM) 

Xerogels were morphologically analyzed using field-emission SEM (Phenom_XL, Alfatest, Milan, Italy). A total of 10 μL of hydrogel was drop-cast and allowed to air dry on an aluminum stub to prepare the samples. For 75 s, a thin layer of palladium and gold was sputtered at a current of 25 mA. Following the introduction of the sputter-coated samples into the specimen chamber, pictures were taken using a secondary electron detector (SED) at an accelerating voltage of 10 kV.

### 4.12. Encapsulation and Release of Naphthol Yellow S

Naphthol yellow S (NYS) encapsulating HGs were prepared in 1.5 mL conical tubes, following the procedure previously described. An NYS water solution was used to rehydrate the DMSO solution of each peptide at a concentration of 0.012 mol L^−1^ (final NYS concentration: 0.011 mol L^−1^). UV-Vis spectroscopy was used to analytically quantify the concentration of NYS solution (ε_430_ = 9922 L·cm^−1^). A total of 800 µL of water was poured over each hydrogel, and 400 µL of this solution was removed and replaced with 400 µL of fresh water at predetermined intervals. UV-vis spectroscopy was carried out to estimate the quantity of NYS in each fraction, which was then expressed as a percentage of the ratio between the original amount that was encapsulated and the released NYS.

### 4.13. Cell Lines

The IRCCS SYNLAB SDN Biobank, Naples, Italy (10.5334/ojb.26, 13 Feb 2017) provided the human aneuploid immortal keratinocyte HaCaT and the mouse pre-adipocyte 3T3-L1 cell lines. The cells were cultured in Dulbecco’s modified Eagle medium with 10% fetal bovine serum and 1% L-glutamine added. Seeds were placed in 100 mm culture dishes and the cells were incubated at 37 °C with 5% CO_2_.

### 4.14. Cell Viability and Survival Test

Both HaCaT and 3T3-L1 cell lines were seeded at a density of 1.5×10^3^ cells per well in 96-well plates for the adhesion test. Each well was filled with 50 µL of the designated hydrogels prior to seeding. Using a light microscope, adhering cells were counted, and the percentage of adherent cells relative to the number of plated cells was determined at increasing times (24, 48, and 72 h). The MTS (3-(4,5-dimethylthiazol-2-yl)-5-(3-carboxymethoxyphenyl)-2-(4-sulfophenyl)-2H-tetrazolium) assay (Cell Titer 96 Aqueous One Solution Cell Proliferation Assay, Promega, Italy) was used to test the toxicity of hydrogels conditioned media. The procedure was carried out according to the manufacturer’s instructions, wherein cells were seeded at a density of 1.5 × 10^3^ HaCaT and 3T3-L1 cells per well in 24-well plates, and the medium was incubated for 24, 48 and 72 h. To obtain the conditioned medium, hydrogels were formulated in a 200 µL hollow plastic chamber [53]. In brief, hydrogels were created in a plastic support that was sealed with a porous membrane at one end. They were then incubated for 16 h at room temperature in sterile conditions with 2 mL of the final mixture. After incubation, there was no noticeable shift in the media’s color, and a pH value of 7.5–7.8 was found to be suitable to culture the cell lines seeded into the wells. The cells were grown in the conditioned media for 24, 48, and 72 h. Following the manufacturer’s recommendations, the MTS test was used every 24 h to determine cell viability [54]. The Victor Nivo Multimode Microplate Reader (PerkinElmer San Jose, USA) was used to examine the samples at an absorbance of 490 nm. The percentage of viable cells in the presence of hydrogels was used to represent cell survival in comparison to that of control cells cultivated without them. Both assays were carried out in triplicate and repeated twice with comparable outcomes.

## Figures and Tables

**Table 1:** **Characterization of pure and mixed hydrogels at different *w*/*w* ratios.** Swelling percentage, gelation time, storage modulus (G′), loss modulus (G″), and Tanδ.

System	Concentration (wt%)	Ratio (*w*/*w*)	Swelling (%)	G′ (Pa)	G″ (Pa)	tanδ	G_t_ (min)
**Fmoc-FFK**	0.5	-	32.4	5.8	1.3	0.224	75
	1.0	-	34.0	15.4	3.6	0.234	45
	2.0	-	37.5	24.3	5.6	0.230	30
**Fmoc-FFK/Fmoc-FF**	1.0	1/1	35.0	315	36	0.114	10
	1.0	1/5	35.1	6930	842	0.121	78
	1.0	1/10	33.8	9800	1195	0.122	138
	1.0	1/20	33.7	9412	495	0.0526	216

## Data Availability

All data and materials are available on request from the corresponding author. The data are not publicly available due to ongoing research.

## Supplementary Materials

The following supporting information can be downloaded at: https://www.mdpi.com/article/10.3390/gels10010012/s1, Figure S1: Physicochemical characterization of Fmoc-FFK peptide; Figure S2: ^1^HNMR spectrum of Fmoc-FFK; Figure S3: Gelation kinetics of gels via UV-vis; Figure S4: SEM microphotos of Fmoc-FFK; Figure S5: CD spectra of mixed Fmoc-FFK/Fmoc-FF HGs; Figures S6–S10: rheological characterization of pure and mixed HGs; Figure S11: release kinetics of NYS from mixed HGs. Table S1: swelling test results. Table S2: A quantitative multivariate percentage analysis for secondary structure estimation (SEE).
