# Supplementary material for: Ultrashort Cationic Peptide Fmoc-FFK as Hydrogel Building Block for Potential Biomedical Applications
Source: Gels. 2023 Dec 22;10(1):12. doi: 10.3390/gels10010012 (PMC10815546; doi:10.3390/gels10010012)
Supplement: Supplementary file 1 [file gels-10-00012-s001.zip › gels-2764328-supplementary.pdf]

Article

Ultrashort Cationic Peptide Fmoc-FFK as Hydrogel Building Block for Potential Biomedical Applications

Enrico Gallo [1], Carlo Diaferia [2], Sabrina Giordano [1], Elisabetta Rosa [2], Barbara Carrese [1], Gennaro Piccialli [2], Nicola Borbone [2], Giancarlo Morelli [2], Giorgia Oliviero [3] and Antonella Accardo [2,*]

[1] IRCCS SYNLAB SDN, Via Gianturco 113, 80143 Naples, Italy; enrico.gallo@synlab.it (E.G.); sabrina.giordano@synlab.it (S.G.); barbara.carrese@synlab.it (B.C.)

[2] Department of Pharmacy, University of Naples "Federico II", Via D. Montesano 49, 80131 Naples, Italy; carlo.diaferia@unina.it (C.D.); elisabetta.rosa@unina.it (E.R.); gennaro.piccialli@unina.it (G.P.); nicola.borbone@unina.it (N.B.); gmorelli@unina.it (G.M.)

[3] Department of Molecular Medicine and Medical Biotechnologies, University of Naples "Federico II", Via S. Pansini 5, 80131 Naples, Italy; giorgia.oliviero@unina.it

* Correspondence: antonella.accardo@unina.it

Supplementary Materials

A)

B)

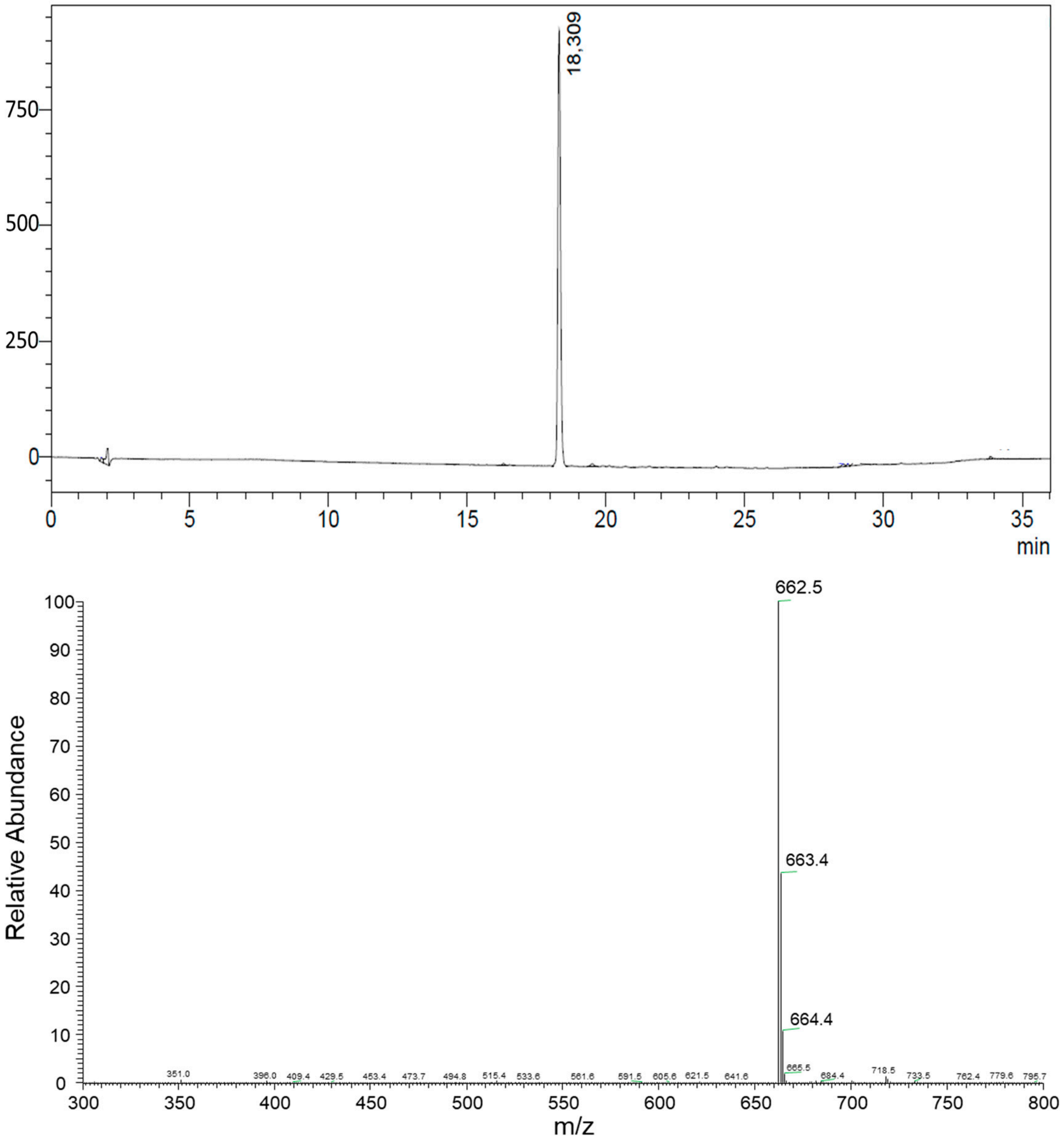

Figure S1: Physicochemical characterization of Fmoc-FFK peptide: A) RP-HPLC chromatography and B) ESI mass spectrum

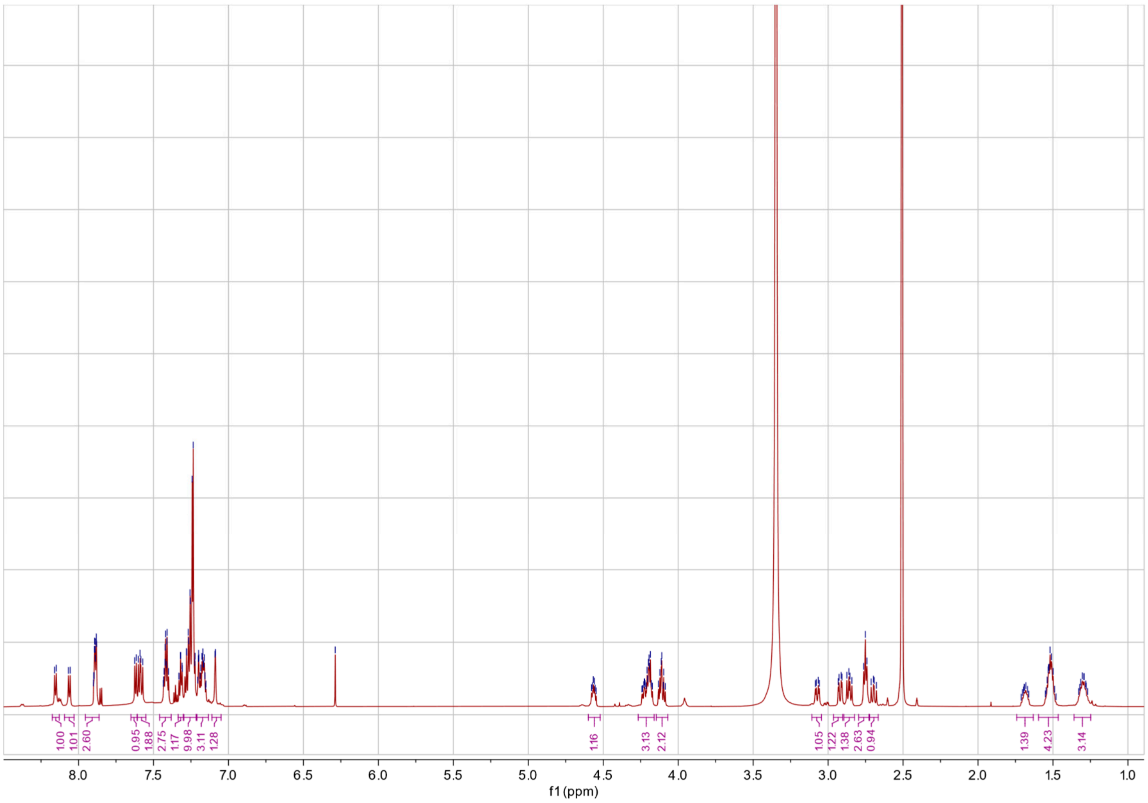

Figure S2: ^{1}HNMR spectrum of Fmoc-FFK.

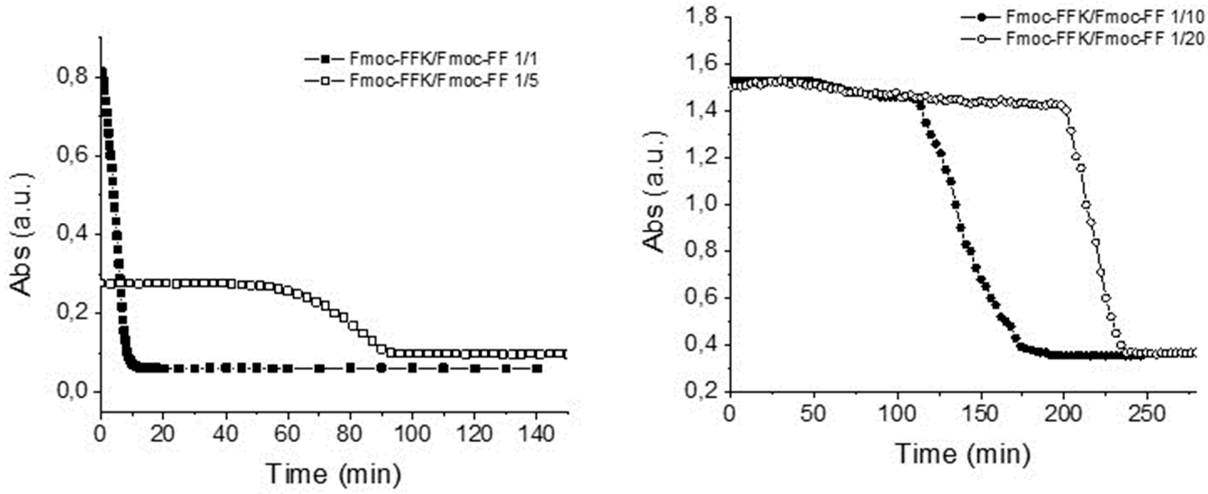

Figure S3: UV-Vis profiles of mixed hydrogels (at different molar ratios) in the long range as function of the time.

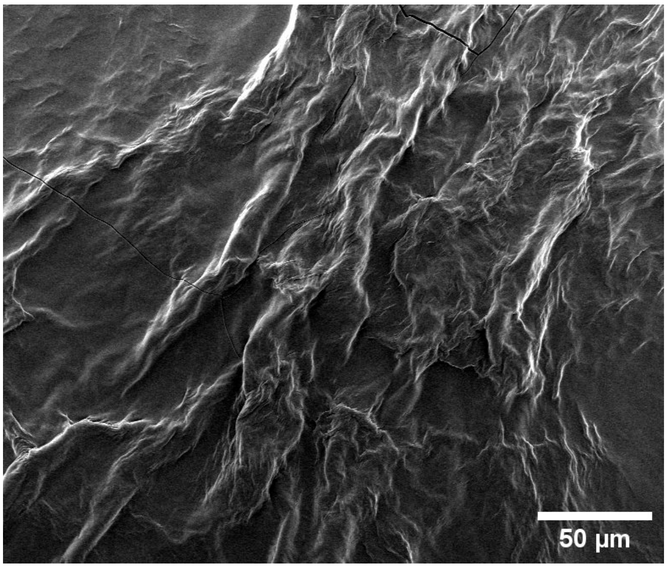

Figure S4: Microphotos of pure xerogels of Fmoc-FFK 2.0 wt %. Scale bar 50 μm.

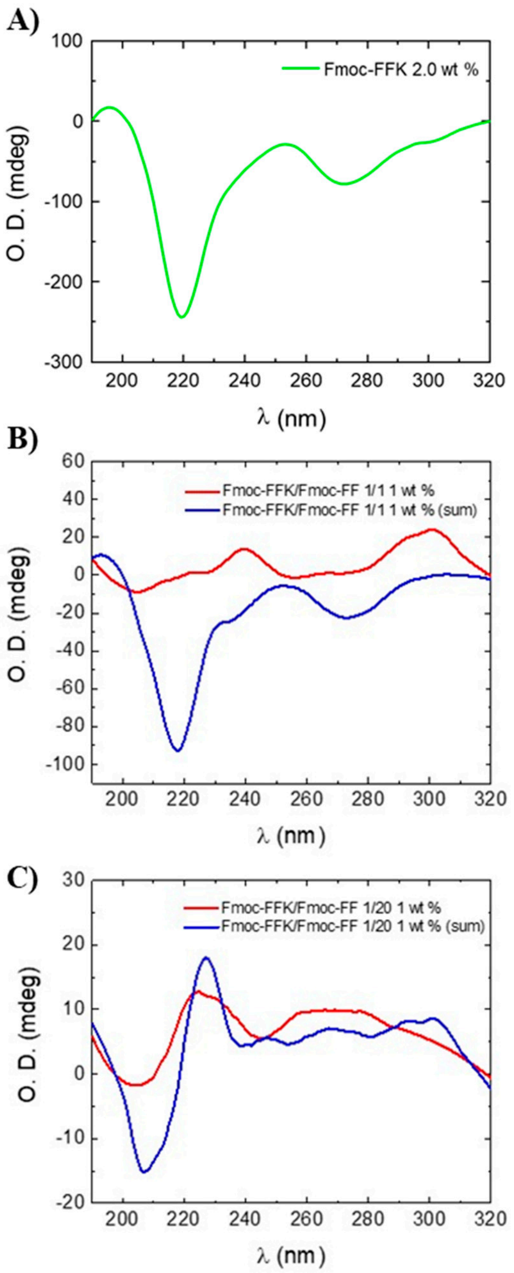

Figure S5: CD spectra in optical density of: A) Fmoc-FFK 2.0 wt%, B) mixed Fmoc-FFK/Fmoc-FF 1/1 at 1.0 wt%, and C) mixed Fmoc-FFK/Fmoc-FF 1/20 at 1.0 wt% (red lines). These spectra of the two mixed hydrogels are reported in comparison to the CD sum of Fmoc-FFK/Fmoc-FF 1/1 and 1/20 (blue lines).

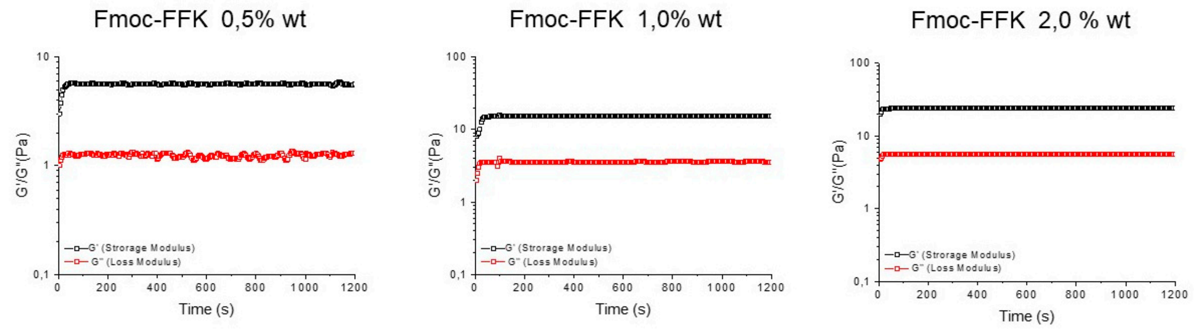

Figure S6: Time sweep (20 minutes) for pure Fmoc-FFK hydrogels at three different concentrations 0.5, 1.0 and 2.0 wt %. Rheological analysis is reported in terms of G' (Storage modulus) and G'' (Loss modulus).

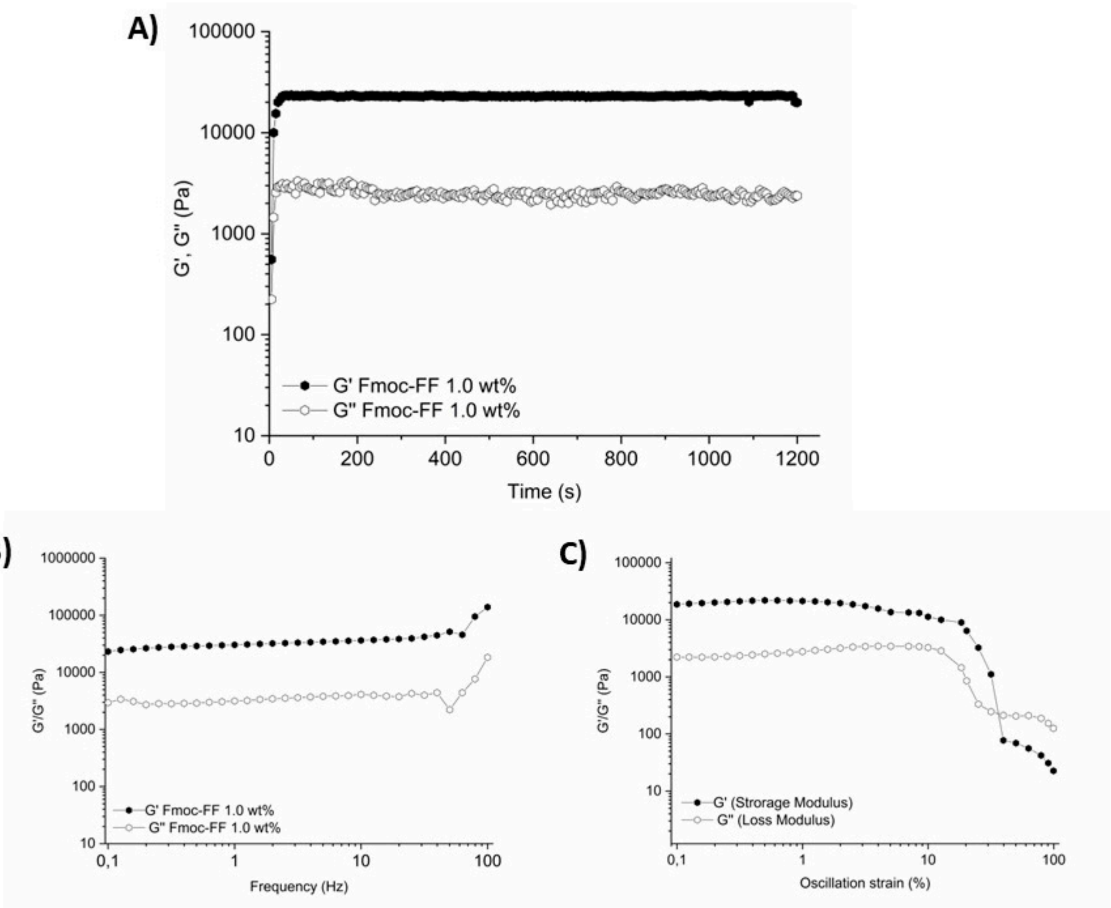

Figure S7: Rheological characterization of self-assembled Fmoc-FF hydrogel: A) time sweep (20 minutes) reported in terms of G' (Storage modulus) and G'' (Loss modulus), B) frequency and C) oscillation strain.

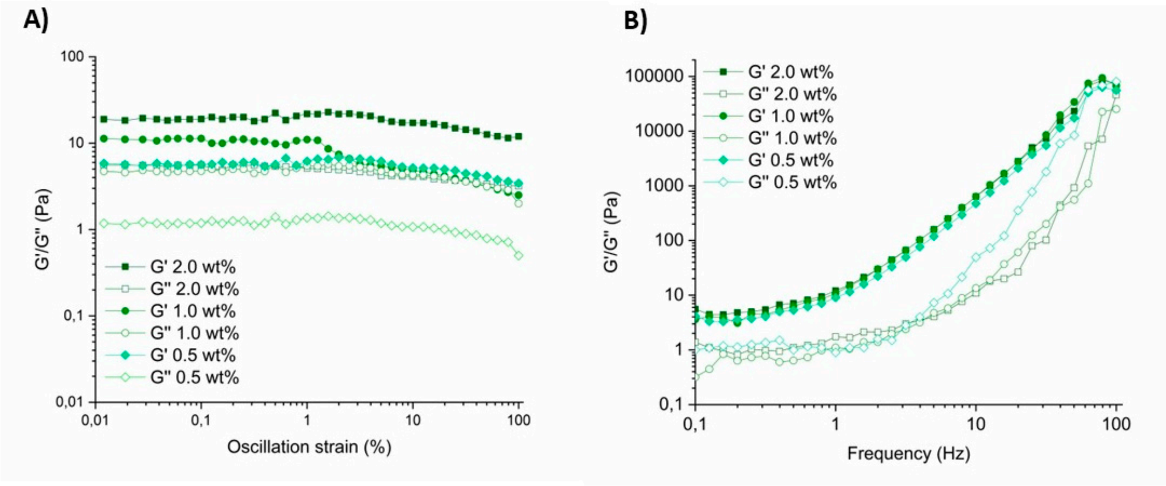

Figure S8: Rheological characterization of self-assembled Fmoc-FFK hydrogels at 0.5wt%, 1.0wt% and 2.0 wt%: A) frequency and B) oscillation strain.

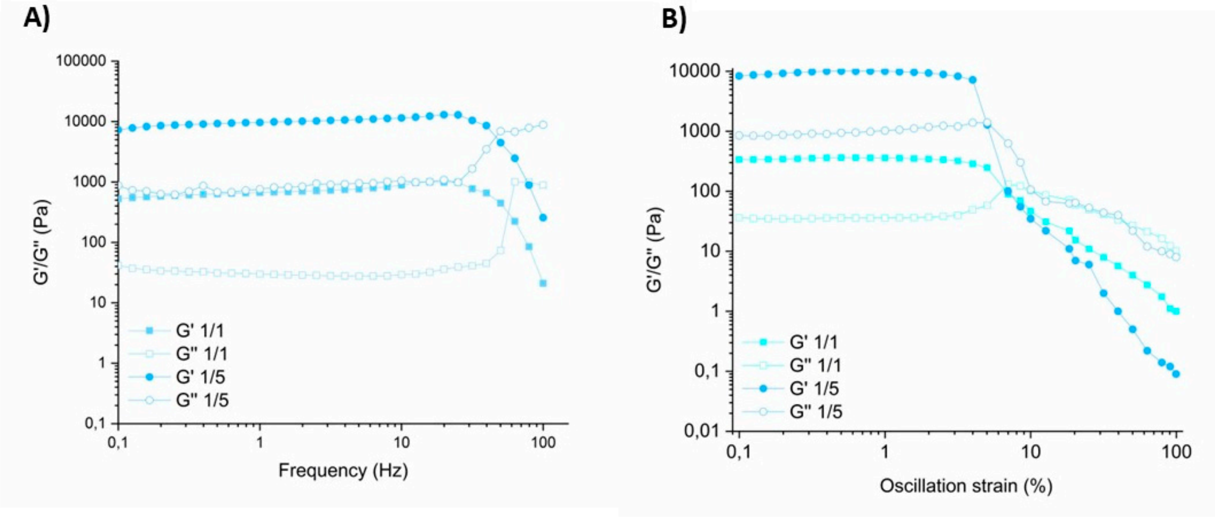

Figure S9: Rheological characterization of mixed Fmoc-FF/Fmoc-FFK hydrogels at 1/1 and 1/5 w/w ratio: A) frequency and B) oscillation strain.

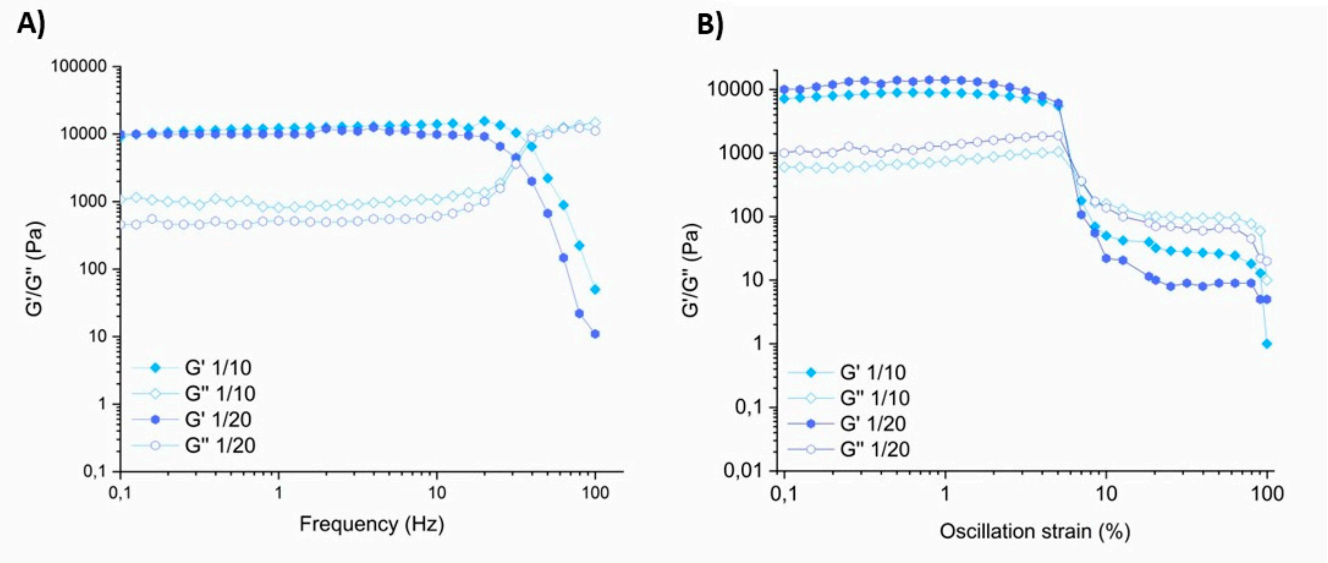

Figure S10: Rheological characterization of mixed Fmoc-FF/Fmoc-FFK hydrogels at 1/10 and 1/20 w/w ratio: A) frequency and B) oscillation strain.

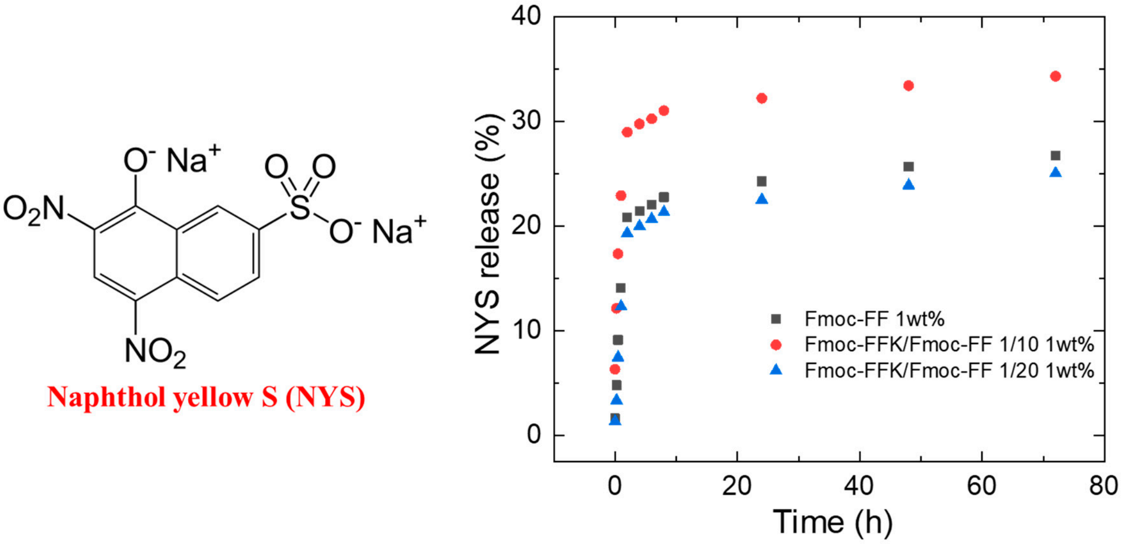

Figure S11: On the left: chemical structure of NYS; on the right: release kinetics of NYS from Fmoc-FF, Fmoc-FFK/Fmoc-FF 1/10 and 1/20 HGs.

Table S1: swelling test results for multicomponent hydrogel Fmoc-FFK/Fmoc-FF in comparison to the other mixed or pure hydrogels from the literature based on Fmoc-FF peptide.

HGs sample	***Concentration (wt%)***	***Ratio (w/w)***	***q %***
Fmoc-FF	1.0	-	29.0
Fmoc-FFK	0.5	-	32.4
	1.0	-	34.0
	2.0	-	37.5
Fmoc-FFK/Fmoc-FF	1.0	1/1	35.0
		1/5	35.1
		1/10	33.8
		1/20	33.7
Fmoc-FFC/Fmoc-FF	1.0	1/5	38.6
		1/10	36.2
		1/20	33.2
Fmoc-FFS/Fmoc-FF	1.0	1/5	34.0
		1/10	34.8
		1/20	36.0
Fmoc-FFT/Fmoc-FF	1.0	1/5	37.0
		1/10	37.9
		1/20	41.0

Table S2: Quantitative multivariate percentage analysis for secondary structure estimation (SEE) achieved *via* method of principal component regression (PCR) with Jasco SEE® dedicated software.

HGs system	***α-helix (%)***	***β-sheet (%)***	***β-turn (%)***	***Others (%)***
Fmoc-FFK 2.0 wt%	0	56	21	23
Fmoc-FF/ Fmoc-FFK 1/1	10	50	19	21
Fmoc-FF/ Fmoc-FFK 1/5	58	12	17	13
Fmoc-FF/ Fmoc-FFK 1/10	66	8	13	13
Fmoc-FF/ Fmoc-FFK 1/20	69	2	12	17
